# Indications and outcomes of liver retransplantation in three medical centers

**DOI:** 10.1590/0100-6991e-20243689-en

**Published:** 2024-06-14

**Authors:** WAGNER AUGUSTO SCHIEL, JULIO CEZAR UILI COELHO ECBC-PR, ANDRE LUIS CONDE WATANABE TCBC-DF, MARCO AURÉLIO RAEDER DA COSTA, ALCINDO PISSAIA

**Affiliations:** 1 - Universidade Federal do Paraná, Departamento de Clínica Cirúrgica do Complexo Hospital de Clínicas - Curitiba - PR - Brasil; 2 - Hospital Nossa Senhora das Graças, Serviço de cirurgia do aparelho digestivo e transplante; 3 - Instituto de Cardiologia do Distrito Federal, Departamento de transplante hepático - Brasília - DF - Brasil

**Keywords:** Liver Failure, Liver Transplantation, Graft Rejection, Transplante, Transplante de Fígado, Rejeição de Enxerto, Cirrose Hepática

## Abstract

**Introduction:**

retransplantation is the only viable treatment for patients with irreversible graft loss. The objective of this study was to analyze the indications and outcomes of liver retransplantation in three medical centers.

**Methods:**

a total of 66 patients who underwent liver retransplantation from September 1991 to December 2021 were included in the study. A retrospective analysis was performed evaluating patients demographic, clinical, primary diagnosis, indications for and time interval to retransplantation, complications and patient survival.

**Results:**

from a total of 1293 primary liver transplants performed, 70 required one or more liver retransplant. The main indication for primary transplant was hepatitis C cirrhosis (21,2%). Hepatic artery thrombosis was the main cause of retransplantation (60,6%), with almost half (46,9%) of retransplants having occurred within 30 days from initial procedure. The average survival time after a repeat liver transplant, was 89,1 months, with confidence interval from 54 to 124,2. The 1-,5- and 10- year survival rate following liver retransplant were 48,4%, 38% and 30,1%, respectively. Male gender, primary non function as the cause for retransplant, prolonged operative time and higher MELD were associated with higher mortality.

**Conclusions:**

operative mortality and morbidity rates of liver retransplantation are higher than those of the first transplantation. Male gender, primary non function, prolonged operative time and higher MELD were associated with less favorable outcomes.

## RESUMO

**Introdução:**

retransplante é o único tratamento viável para pacientes com perda irreversível do enxerto. O objetivo deste estudo foi analisar as indicações e resultados do retransplante hepático em três centros médicos.

**Métodos:**

foram incluídos no estudo 66 pacientes submetidos a retransplante hepático no período de setembro de 1991 a dezembro de 2021. Foi realizada uma análise retrospectiva avaliando dados demográficos, clínicos, diagnóstico primário dos pacientes, indicações e intervalo de tempo para retransplante, complicações e sobrevida do paciente.

**Resultados:**

de um total de 1.293 transplantes primários de fígado realizados, 70 necessitaram de um ou mais retransplantes de fígado. A principal indicação de transplante primário foi cirrose por hepatite C (21,2%). A trombose da artéria hepática foi a principal causa de retransplante (60,6%), sendo que quase metade (46,9%) dos retransplantes ocorreu dentro de 30 dias do procedimento inicial. O tempo médio de sobrevivência após retransplante de fígado foi de 89,1 meses, com intervalo de confiança de 54 a 124,2. A taxa de sobrevivência de 1,5 e 10 anos após o retransplante de fígado foi de 48,4%, 38% e 30,1%, respectivamente. Gênero masculino, disfunção primária do enxerto como causa de retransplante, tempo operatório prolongado e maior MELD foram associados a maior mortalidade.

**Conclusão:**

as taxas de mortalidade e morbidade operatórias do retransplante hepático são superiores às do primeiro transplante. Sexo masculino, disfunção primária do enxerto, tempo operatório prolongado e maior MELD foram associados a desfechos menos favoráveis.

## INTRODUCTION

Despite significant advances in surgical technique, patient management and immunosuppressive protocols, graft loss occurs in 5 to 20% of liver transplant recipients^1^ ^-^ ^4^. Liver retransplantation (re-LT) represents the only effective treatment option for patients who suffer graft loss following primary liver transplantation (LT). Indications of re-LT comprise vascular complications, graft infarction, acute and chronic rejection, primary non-function, recurrence of the primary liver disease, among others^1^ ^,^ ^2^. 

Being a major technical and surgical challenge, liver retransplant also poses ethical, social and clinical concerns due to its significant poorer outcome when compared to a first liver transplant^3^ ^-^ ^5^. Considering the growing shortage of organ donors, the decision for re-LT may be difficult due to the inferior results when compared to the allocation of the organ to another patient who would receive the first liver transplant. An additional aspect concerns the increasing use of marginal grafts. This could also increment graft loss, and therefore raising the need of retransplant. 

Although several reports on re-LT have been published, most of them are based on small sample sizes^5^ ^-^ ^7^. There is also a lack of consensus and guidelines to help the decision-making process in the indication of re-LT. Latin America literature on this subject is scarce, with only a few manuscripts published in Brazil. The objective of this study is to present the indications and outcomes of re-LT in three major transplant centers in Brazil. 

## METHODS

We performed a retrospective analysis of patients who underwent LT in three Brazilian medical Hospitals from September 1991 to December 2021. The institutions that participated in the study were the Hospital de Clínicas of the Federal University of Paraná, Hospital Nossa Senhora das Graças of Curitiba, and Instituto de Cardiologia do Distrito Federal of Brasília. Data of all patients were collected from electronic medical records and study protocols. The protocol of this study was approved by the Ethics Committee of the University Hospital of the Federal University of Paraná, Brazil (CAAE 40183120.4.1001.0096).

LT was performed using standard surgical techniques, previously described^8^ ^-^ ^10^. After LT, patients were placed on standard immunosuppressive protocol consisting of tacrolimus or cyclosporine, azathioprine or mycophenolate mofetil, and prednisone.

### Data Analysis

The following variables were analyzed: demographic data, primary diagnosis and indication of re-LT, time interval between first and repeat liver transplant, preoperative MELD score, transplant technique, graft function and complications. The overall patient survival rates were calculated and stratified in periods of time. The study also analyzed several variables in order to determine those involved with survival. Post-operatory complications were assessed with the Clavien-Dindo Scoring System^11^.

### Statistical Analysis

Quantitative variables were described by standard deviation, median, mean, minimum and maximum values. Qualitative variables were presented in frequency and percentage. The Kaplan-Meier estimates for survival after first liver transplant and retransplant (in months) were shown in tables, survival graphics and mean survival estimates with their corresponding confidence intervals. For the analysis of factors associated with mortality after the first retransplantation, univariate Cox Regression models were adjusted with the significance level of the Wald test.

The factors which presented a significance level lower than 10% in the Wald test of the univariate models were adjusted in a multiple Cox regression model with selection of variables by the Backward Stepwise (Wald) method. The estimated association measure was hazard ratio (HR), with a confidence interval of 95%. Also, the duration of operation for both transplant and retransplant were compared using the Student’s t-test.

Values of p<0,05 indicated statistical significance. The data were analysed with the software IBM SPSS Statistics v.28.0. Armonk, NY: IBM Corp. No values were imputed to correct absent data. 

## RESULTS

From September 1991 to December 2021, 1293 patients underwent liver transplant in the three transplantation centers. Seventy of these patients were subjected to retransplants, 5.4 % of total transplants performed. Four retransplanted patients were not included in the analysis due to lack of complete data. Six patients were subjected to 2 liver retransplants. Only the data of the first retransplantation was considered for analysis. Of the 66 patients included in the study, 56 (90.3%) underwent deceased donor liver transplant and 6 (9.7%) a living donor liver transplant. Four did not have data on liver donor type. Most patients were male (N=52; 80%). The mean age of the patients were 43,1 ± 17,4 years (range 4 - 69 years). The demographic characteristics of the patients are shown in Table 1. 

**Table 1:** Demographic characteristics of the 66 patients who were subjected to liver retransplant.

Demographic Characteristics	Classification	Result*
Age at Retransplant mean± SD; median (range)		43,1 ± 17,4; 47 (4 - 69)
Gender	M	52 (80%)
Time between LT and Retransplantation (days)		394 ± 831,6; 34,5 (2 - 4389)
Interval between LT and Retransplantation	≤30 days	30 (46,9%)
	>30 days	34 (53,1%)
BMI		23,7 ± 4,1; 23,6 (15 - 32,2)
BT (ABO)	A	24 (68,6%)
	B	2 (5,7%)
	O	9 (25,7%)
MELD at Retransplantion		32,3 ± 12,5; 31 (6 - 56)
HCC	Yes	18 (34%)
Smoking	Yes	18 (29,5%)
Alcohol use	Yes	18 (29,5%)
Previous Surgery	Yes	19 (31,7%)

*Categorical variables were described by frequency (percentage) and quantitative variables were described by mean ± standard deviation; median (minimum value - maximum value). LT: Liver Transplant (Primary); ReLT: Repeat Liver Transplant; SD: Standard Deviation; BMI: Body Mass Index; BT: Blood Type; HCC: Hepatocellular Carcinoma.

The indications of the first liver transplantation are shown in Table 2. The main indications were hepatitis C cirrhosis (21,2%), alcoholic liver disease (18,2%), cryptogenetic (10,6%), autoimmune hepatitis and hepatitis B cirrhosis (9,1% each), followed by NASH (7,6%), primary sclerosing cholangitis (6,1%), primary biliary cirrhosis (3%) and fulminant hepatitis (3%). Associated hepatocellular carcinoma was diagnosed in 18 (34%) patients.

**Table 2:** Clinical characteristics of the 66 patients who were subjected to liver retransplant.

Clinical Characteristics	Classification	Result*
Main Etiology - Liver disease	Alcohol	12 (18,2%)
	NASH	5 (7,6%)
	HCV	14 (21,2%)
	HBV	6 (9,1%)
	AIH	6 (9,1%)
	FUL HEP	2 (3%)
	PSC	4 (6,1%)
	PBC	2 (3%)
	Criptogenetic	7 (10,6%)
	Other	8 (12,1%)
Type of LT	Cadaveric	56 (90,3%)
	Living-donor	6 (9,7%)
Time on waiting list (days)		132,8 ± 233; 54 (1 - 1206)
Operation Time (min)		418,2 ± 95; 420 (200 - 660)
Patient survival after primary LT (months)		51,2 ± 70,5; 22,5 (0,1 - 306,8)

*Categorical variables were described by frequency (percentage) and quantitative variables were described by mean ± standard deviation; median (minimum value - maximum value). LT: Liver Transplant (Primary); ReLT: Repeat Liver Transplant; NASH: Nonalcoholic Steatohepatitis; HCV: C-Virus Hepatitis; HBV: B-Virus Hepatitis; AIH: Autoimmune Hepatitis; FUL HEP: Fulminant Hepatitis; PSC: Primary Sclerosing Cholangitis; PBC: Primary Biliary Cirrhosis.

The main indication for retransplant was hepatic artery thrombosis (HAT) (n=40; 60.6%). Other causes included primary non-function (PNF) (n=13; 19.7%), chronic rejection (n=10;15.2%), recurrence of the initial disease (n=2; 3%), and ischemic cholangitis (n=1; 1.5%). 

The mean time interval between the first liver transplant and the retransplant was of 394 ± 831,6 days (range 2 - 4389). Thirty patients (46,9%) had undergone a retransplant within 30 days from the initial procedure.

The mean preoperative MELD score was 32,3 ± 12,5 (range 6 - 56). The mean cold ischemia time was 320,8 ± 161,3 (range 45 - 810) minutes. The mean operative time was 385,4 ± 111,2 (range 176 - 750) minutes. Hospital stay duration was 20,5 ± 20,7 (1 - 135) days. Time on waiting list was 38,1 ± 78,5 (range 1 - 439) days for the re-LT. 

The analysis of donor characteristics revealed a predominance of male gender (n=17; 56.7%). The mean age was 36,7 ± 16,4 years.

### Survival after liver transplant

**Figure 1:**
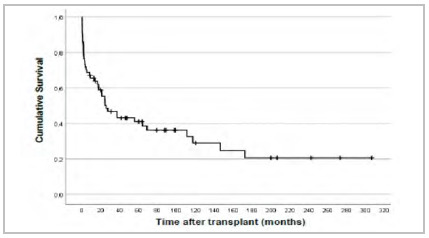
Survival after liver transplant.

The Kaplan-Meier estimates for the proportion of survivors after liver transplant are shown in Figure 1. Of the 64 cases considered, 42 died and 22 remain alive. 

The average survival time after a liver transplant, considering the follow-up period of 300 months, was 95,3 months, with a confidence interval from 61,5 to 129,2. Cumulative survival following primary liver transplant is represented in Figure 1.

### Survival after retransplantation

**Figure 2:**
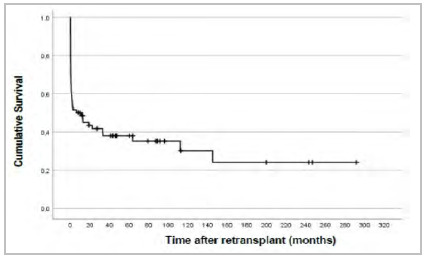
Survival after retransplant.

The Kaplan-Meier estimates for the proportion of survivors following a liver retransplant are shown in Figure 2. Of the 66 cases considered, 43 died and 23 are still alive.

The average survival time after a repeat liver transplant, considering a follow-up time of 291 months, was 89,1 months, with confidence interval from 54 to 124,2. The 1-,5- and 10- year survival rate following liver retransplant were 48,4%, 38% and 30,1%, respectively.

Cumulative survival curves following retransplant are shown in Figure 2.

### Analysys of factors associated with mortality after retransplant

#### Univariate Analysis

For each of the analyzed variables, the null hypothesis that there is no association between the variable and progression-free time (time elapsed until progression) was tested versus the alternative hypothesis that there is an association. For this purpose, Cox Regression models were adjusted and hazard ratio (HR) values ​​were estimated with respective 95% confidence intervals.

#### Multiple analysis

A multiple model of analysis was set to determine the instantaneous probability of mortality following a repeat liver transplant. In this analysis, were included the variables with significance lower than 0,10 in Wald’s univariate test, in addition to the patient’s prior Meld score, given its well stablished correlation with mortality. Given the absence of data on “Number of packed red blood cells in retransplantation” in 14 patients, a model without this variable was adjusted, in order to determine a larger sample size and statistical power. Table 7 presents the multiple complete Cox regression models and the best adjusted parameters given the insertion of data by the Backward Stepwise method (Wald).

[Table t3]

**Table 3 t3:** Technical aspects of the liver retransplants.

Variable	Classification	Resultado*
Technique	Cava-caval	22 (66,7%)
	Piggyback	11 (33,3%)
Biliary Anastomosis	Roux-en-y	15 (46,9%)
	TT	17 (53,1%)
Warm ischemia time (min)		50 ± 24,4; 45 (24 - 165)
Cold ischemia time (min)		320,8 ± 161,3; 300 (45 - 810)
Total ischemia time (min)		402,5 ± 154,7; 406 (160 - 673)
Operation Time (min)		385,4 ± 111,2; 379 (176 - 750)

*Categorical variables were described by frequency (percentage) and quantitative variables were described by mean ± standard deviation; median (minimum value - maximum value). LT: Liver Transplant (Primary); ReLT: Repeat Liver Transplant; TT: Termino-terminal.

[Table t4]

**Table 4 t4:** Indications and results of the 66 liver retransplants.

Variable	Classification	Resultado*
Main Cause	HAT	40 (60,6%)
	PNF	13 (19,7%)
	Chronic Rejection	10 (15,2%)
	Relapse	2 (3%)
	Other	1 (1,5%)
Length of Hospital Stay (days)		20,5 ± 20,7; 16 (1 - 135)
Current Status	Alive	23 (34,8%)
	Deceased	43 (65,2%)
1^st^ Graft Survival Time (months)		13,1 ± 27,7; 1,2 (0,1 - 146,3)
2^nd^ Graft Survival Time (months)		33,9 ± 62,3; 4,1 (0 - 291,1)
Patient survival after ReLT (months)		37,6 ± 63,8; 7,2 (0 - 291,1)

*Categorical variables were described by frequency (percentage) and quantitative variables were described by mean ± standard deviation; median (minimum value - maximum value). LT: Liver Transplant (Primary); ReLT: Repeat Liver Transplant; HAT: Hepatic Artery Thrombosis; PNF: Primary Non Function.

[Table t5]

**Table 5 t5:** Surgical complications of the 66 patients who were subjected to liver retransplant.

Variable	Classification	Result*
Clavien-dindo Score	1, 2 or 3	25 (39,7%)
	3b, 4 or 5	38 (60,3%)
Intraoperatory Bleeding	No	49 (74,2%)
	Yes	17 (25,8%)
Biliary Stenosis	No	57 (86,4%)
	Yes	9 (13,6%)
Incisional Hernia	No	62 (93,9%)
	Yes	4 (6,1%)
Sepsis	No	49 (74,2%)
	Yes	17 (25,8%)
Refractary Shock	No	52 (78,8%)
	Yes	14 (21,2%)
Reoperation	No	45 (68,2%)
	Yes	21 (31,8%)
Variable	Classification	Result*
Biliary fistula	No	65 (98,5%)
	Yes	1 (1,5%)
CMV Infection	Yes	21 (48,8%)
Intraoperative Blood Transfusion	No	10 (17,2%)
	Yes	48 (82,8%)
No. Of RBC Concentrates		4,2 ± 4,5; 2 (0 - 21)

*Categorical variables were described by frequency (percentage) and quantitative variables were described by mean ± standard deviation; median (minimum value - maximum value). LT: Liver Transplant (Primary); ReLT: Repeat Liver Transplant; CMV: Cytomegalovirus; RBC: Red Blood Cell.

[Table t6]

**Table 6 t6:** Factors associated with increase in mortality in patients undergoing liver retransplantation.

Factors	Classification	Descriptive Result of Mortality*	HR (CI of 95%) for Mortality^#^	p-value^§^
Sex	Female	4 (30,8%)	Ref.	
	Male	39 (73,6%)	3,126 (1,126 - 8,931)	0,029
Age at Retransplant	Alive	40,2 ± 16,9; 42 (14 - 67)		
	Deceased	44,7 ± 17,7; 49 (4 - 69)	1,016 (0,997 - 1,034)	0,099
Meld at Retransplant	Alive	32,4 ± 11,9; 30 (12 - 56)		
	Death	32,3 ± 12,9; 31 (6 - 56)	1,017 (0,987 - 1,049)	0,265
Main Etiology	Alcohol	6 (50%)	Ref.	
	NASH	4 (80%)	2,794 (0,782 - 9,986)	0,114
	CHV	11 (78,6%)	2,021 (0,744 - 5,49)	0,167
	BHV	4 (66,7%)	1,528 (0,424 - 5,5)	0,517
	AIH	1 (16,7%)	0,2 (0,024 - 1,676)	0,138
	FUL HEP	1 (50%)	0,586 (0,07 - 4,917)	0,622
	PSC	3 (75%)	1,056 (0,261 - 4,268)	0,939
	PBC	1 (50%)	1,21 (0,145 - 10,08)	0,860
	Cryptogenic	6 (85,7%)	2,112 (0,68 - 6,564)	0,196
	Other	6 (75%)	1,838 (0,587 - 5,757)	0,296
ReLT Main Cause	HAT	22 (55%)	Ref.	
	Graft Failure	10 (76,9%)	2,361 (1,110 - 5,023)	0,026
	Chronic Rejection	8 (80%)	1,398 (0,617 - 3,170)	0,422
	Relapse/other	3 (100%)	4,242 (1,249 - 14,405)	0,021
Time between LT and ReLT	≤30 days	17 (56,7%)	Ref.	
	>30 days	25 (73,5%)	1,337 (0,721 - 2,481)	0,357
Clavien-dindo	1, 2 or 3	16 (64%)	Ref.	
	3b, 4 or 5	27 (71,1%)	1,216 (0,654 - 2,263)	0,537
Time on waiting list (days)	Survival	31,2 ± 71,8; 6 (1 - 290)		
	Death	42,1 ± 82,8; 6,5 (1 - 439)	1,001 (0,997 - 1,005)	0,702
Cold Ischemia Time (min)	Survival	293,7 ± 109,6; 255 (207 - 541)		
	Death	334,4 ± 183,1; 342,5 (45 - 810)	1,002 (0,999 - 1,006)	0,198
Operatory Time (min)	Survival	356,4 ± 99,4; 355 (210 - 570)		
	Death	402,1 ± 115,4; 404 (176 - 750)	1,003 (1 - 1,006)	0,035
Factors	Classification	Descriptive Result of Mortality*	HR (CI of 95%) for Mortality^#^	p-value^§^
Nº of RBC concentrates	Survival	3,4 ± 2,7; 2,5 (0 - 10)		
	Death	4,7 ± 5,2; 2 (0 - 21)	1,074 (0,997 - 1,156)	0,059
Donor Age	Survival	32,4 ± 16; 22 (17 - 57)		
	Death	38,6 ± 16,5; 40 (14 - 65)	1,006 (0,981 - 1,031)	0,639

*Quantitative variables were described by their mean values ± standard deviation; median (minimum value - maximum value) and categorical variables described by frequency (percentage) of deaths for the total in the line. #Hazard ratio (HR) and confidence interval of 95% (CI de 95%) for mortality, from univariate Cox’s regression models. §Wald’s test level of significance, p<0,05. NASH: Nonalcoholic Steatohepatitis; HCV: C-Virus Hepatitis; HBV: B-Virus Hepatitis; AIH: Autoimmune Hepatitis; FUL HEP: Fulminant Hepatitis; PSC: Primary Sclerosing Cholangitis; PBC: Primary Biliary Cirrhosis; HAT: Hepatic Artery Thrombosis; RBC: Red Blood Cell.

**Table 7:** Multiple cox regression model adjusted for mortality

Stages Of the model	Factors	Model adjusted for mortality without the factor “number of rbc concentrates”	
		HR (CI de 95%)^#^	P-value^§^
Complete model (stage 01)	Male Sex (ref. female)	7,178 (1,376 - 37,449)	0,019
	Age at retransplant	1,021 (0,993 - 1,05)	0,152
	Meld at retransplant	1,047 (1,003 - 1,093)	0,036
	Retransplant - Main Cause (ref. HAT)		
	Graft Failure	4,273 (1,545 - 11,82)	0,005
	Chronic Rejection	1,246 (0,41 - 3,792)	0,698
	Relapse/other	1,597 (0,264 - 9,672)	0,610
	Retransplant - Operatory Time (min)	1,006 (1,002 - 1,011)	0,010
	Retransplant - Number of RBC	-	-
Best adjusted model	Male Sex (ref. female)	8,592 (1,709 - 43,205)	0,009
	Age at Retransplant	-	-
	Meld at Retransplant	1,051 (1,009 - 1,095)	0,018
	Retransplant - Main Cause (ref. HAT)		
	Graft Failure	4,132 (1,522 - 11,218)	0,005
	Chronic Rejection	1,314 (0,444 - 3,887)	0,622
	Relapse/Other	1,674 (0,277 - 10,106)	0,574
	Retransplant - Operatory Time (min)	1,007 (1,002 - 1,011)	0,005

#Hazard ratio (HR) and Confidence Intervals of 95% (CI of 95%) for mortality, from Cox multiple Regression model with entrance of variables through Backward Stepwise (Wald) model. §Wald’s level of significance, p<0,05. HAT: Hepatic Artery Thrombosis; RBC: Red Blood Cell.

Table 8 shows the comparison of mean operatory time for transplant and retransplant. No significant statistical difference was observed.

**Table 8:** Comparison of the mean operatory time for transplant and retransplant.

VARIABLE	MOMENT	VALID n	MEAN ± SD	AVERAGE DIFFERENCE (IC de 95%)	p-value
OPERATORY TIME (min)	Transplant	56	414,6 ± 94,5	34,09 (-0,08; 68,25)	0,050*
	Retransplant		380,5 ± 112,3		

SD: Standard Deviation; CI of 95%: Confidence Interval of 95%. *Student’s t-test’s level of significance for paired samples.

## DISCUSSION

This study represents the findings of patients subjected to re-LT in three liver transplantation centers in two different regions in Brazil. Our liver retransplantation rate (5.4%) is comparable to that reported in most studies from other institutions (4.8 to 22%)^7^ ^,^ ^12^ ^-^ ^17^. These rates were possibly underestimated, since they included only patients that actually underwent liver retransplant, excluding those who died on the waiting list. The lack of well-defined criteria for retransplant in Brazil might also have contributed to the lower rate of retransplant in our study.

Contrary to some reports, in the present study, the transplants were not divided according to their time period, due to the difference in experience among the three transplant centers. In addition, transplants in the pre-MELD era were performed in only one of the transplant centers. The data for MELD scores on this subset of patients was either adjusted according to available data or excluded from the analysis. In Brazil, before the advent of MELD score in 2006, organ allocation was chronological, based mostly on time spent on waiting list, with few situations conferring high-urgency status. Patients with liver failure after graft failure were prioritized to receive another organ, by enhancing MELD status up to 40 in those patients listed within 7 days from the initial procedure. In this study, a higher MELD value was proved to be an independent risk factor for mortality in patients following a liver retransplant, as pointed out in several studies^13^ ^,^ ^18^. A Brazilian cohort also showed no difference in mortality with respect to MELD scores in retransplanted patients^12^. 

The main indication for both early and late retransplant was hepatic artery thrombosis (HAT), followed by primary non-function (PNF) and ductopenic rejection. The incidence of HAT in this study was superior to that shown in other studies, which varied from 11.5 to 40% of all retransplant cases in adults^13^ ^,^ ^16^ ^,^ ^19^ ^-^ ^22^. In addition, it does not reflect the total incidence of HAT in transplanted patients, since it does not contemplate those who had HAT and underwent an interventional procedure other than retransplant, not included in this study. The higher incidence of HAT could be attributed to several factors, such as, older and marginal donors^23^ ^,^ ^24^. The incidence of PNF in our study (18.2%) was similar to those of other reports (10 to 32.3%)^16^ ^,^ ^20^ ^-^ ^22^.

As observed in our study, several authors have also shown that the operative mortality and morbidity rates of liver retransplantation are higher than those of the first transplantation. Retransplantation is usually a procedure of great technical complexity due to the presence of extensive and firm adhesions, and severe graft disfunction/failure. The duration of the operation and the operative bleeding are extensive, even when LT is performed in a referral center. 

Our retransplantation survival rate was lower than the reported in the United States and Europe, possibly due to several medical limitations of developing countries, such as Brazil, including shortage of appropriate hospital resources and patients’ economic and cultural differences. However, our survival rate was similar to that reported by other Brazilian centers^12^ ^,^ ^25^. Despite inferior results compared to primary transplant, liver retransplant is still a suitable and sometimes the only option for patients with a failing graft, recurrence of liver disease and consequently poorer quality of life^8^ ^,^ ^9^.

The assessment of post-operatory complications revealed that 60,3% of retransplanted patients underwent a second interventional procedure (Clavien-Dindo IIIb, IV or V) such as laparotomy, percutaneous drainage, ERCP, or either shock or death. Although comparable to other studies 26, this incidence is higher since it includes post-operatory deaths (Clavien-Dindo V).

Our study has shown that male gender, PNF as cause of ReLT, higher MELD values and operatory time are independent predictors of mortality in patients following liver retransplant. The cause of ReLT has not been confirmed as predictor of prognosis in the majority of studies^4^ ^,^ ^5^ ^,^ ^27^. In agreement with Marudanayagam et al. (2010), we found that PNF as indication of ReLT had a poorer prognosis^13^. It is important to point out that MELD levels had no significant association with the outcome in the univariate analysis, but had in the multiple analysis, which was not observed with the other variables analyzed. Although operative time was involved with poorer prognosis, no significant difference in duration of operation between primary and repeat liver transplant was observed. However, a trend towards longer operative time was recorded on the retransplant group. Regarding the differences between recipient gender, it is important to highlight that they were not adjusted by age, neither compared according to the gender of the donor. A study by Simone et al. (2020) revealed that female recipients ≥45 years had better outcomes than males of the same age when receiving grafts from female donors^28^.

The major limitation of our study is the retrospective evaluation of the data, which limit a more robust and consistent analysis, despite the large number of patients included. However, this was minimized because the data in our series were retrieved from electronic medical records and study protocols. In addition, prospective studies are difficult to be implemented due to the emergency nature of most retransplantations. 

An important aspect of our study is the lack of studies on ReLT in patients in South America^29^. There are only a few Brazilian publications^12^ ^,^ ^19^ ^,^ ^25^. As results vary across the world regions, our study may be a valuable contribution to this important issue. 

## CONCLUSION

It is concluded from the present study that the main indications of liver retransplantation are hepatic artery thrombosis, liver primary non-function, and ductopenic rejection. Operative mortality and morbidity rates of liver retransplantation are higher than those of the first transplantation. Male gender, primary non function, longer operatory time and higher meld were associated with higher retransplant mortality.
